# Heterologous expression of the yeast Tpo1p or Pdr5p membrane transporters in Arabidopsis confers plant xenobiotic tolerance

**DOI:** 10.1038/s41598-017-04534-7

**Published:** 2017-07-03

**Authors:** Estelle Remy, María Niño-González, Cláudia P. Godinho, Tânia R. Cabrito, Miguel C. Teixeira, Isabel Sá-Correia, Paula Duque

**Affiliations:** 1 Instituto Gulbenkian de Ciência, 2780-156 Oeiras, Portugal; 2 Institute for BioEngineering and Biosciences (iBB), Department of Bioengineering, Instituto Superior Técnico, Universidade de Lisboa, 1049-001 Lisbon, Portugal

## Abstract

Soil contamination is a major hindrance for plant growth and development. The lack of effective strategies to remove chemicals released into the environment has raised the need to increase plant resilience to soil pollutants. Here, we investigated the ability of two *Saccharomyces cerevisiae* plasma-membrane transporters, the Major Facilitator Superfamily (MFS) member Tpo1p and the ATP-Binding Cassette (ABC) protein Pdr5p, to confer Multiple Drug Resistance (MDR) in *Arabidopsis thaliana*. Transgenic plants expressing either of the yeast transporters were undistinguishable from the wild type under control conditions, but displayed tolerance when challenged with the herbicides 2,4-D and barban. Plants expressing *ScTPO1* were also more resistant to the herbicides alachlor and metolachlor as well as to the fungicide mancozeb and the Co^2+^, Cu^2+^, Ni^2+^, Al^3+^ and Cd^2+^ cations, while *ScPDR5*-expressing plants exhibited tolerance to cycloheximide. Yeast mutants lacking Tpo1p or Pdr5p showed increased sensitivity to most of the agents tested in plants. Our results demonstrate that the *S. cerevisiae* Tpo1p and Pdr5p transporters are able to mediate resistance to a broad range of compounds of agricultural interest in yeast as well as in Arabidopsis, underscoring their potential in future biotechnological applications.

## Introduction

Along decades, the development of human activities such as industry has led to the release of large amounts of toxic substances into the environment in the form of organic pollutants and heavy metals. Despite the urgent need to remove toxic substances from natural environments due to the risk they pose to wild life and to human health, current strategies to decontaminate soils are expensive and of low effectivity, normally involving the disposal of at least part of the soil in storage facilities^1^. Although some plant species are able to remove soil pollutants or at least to reduce their hazard, this ability having being exploited in a strategy called phytoremediation^1^, many of the plant species of economic importance (e.g. crops) are not naturally tolerant to the toxic effects of soil pollutants. The quest for strategies to make plants more resilient to such effects is therefore imperative.

The heterologous expression of proteins known to mediate resistance to xenobiotics in their native organisms in other, more sensitive, species has proved a successful means of enhancing plant resistance to toxic substances. For instance, the expression of OYE2, a dehydrogenase from *Saccharomyces cerevisiae*, in *Arabidopsis thaliana* led to an increased plant tolerance to 2,4,6-Trinitrotoluene (TNT) and a higher capacity of removing the compound from the media^2^. An engineered bacterial enzyme, dicamba monooxigenase (DMO), was shown to confer resistance to the herbicide dicamba in tobacco, tomato, Arabidopsis, and soybean plants^3^, thus representing a potential tool for weed resistance management in crops.

Among the types of proteins used for heterologous expression purposes, transporters have shown to be particularly suitable. Arabidopsis and tobacco plants expressing a wheat vacuolar H^+^-pyrophosphatase, TaVP1, were more tolerant to Cd^2+^ and accumulated more of the cation than their wild-type counterparts^4, 5^. Expression of AtHMA4, a P1B-ATPase, or of its C-terminus in tobacco also modified Cd^2+^ as well as Zn^2+^ root/shoot partitioning and tolerance^6^. A similar effect was observed regarding arsenic resistance when the yeast proton gradient-driven antiporter *ScACR3* gene was introduced into Arabidopsis^7^. In yet another example, two Multidrug Resistance-associated Proteins (MRPs) belonging to the ABC superfamily of transporters from *S. cerevisiae*, Ycf1p and Vmr1p, conferred Cd^2+^ and Pb^2+^ resistance to transgenic poplar plants^8^.

The generation of transgenic plants bearing resistance to multiple xenobiotics can easily require several genetic modifications, and thus the identification of single genes conferring Multiple Drug Resistance (MDR) in plant systems is highly desirable. MDR is in most cases conferred by multidrug transporters of the ATP-Binding Cassette Superfamily (ABC) and Major Facilitator Superfamily (MFS). Among the ABC and MFS MDR transporters in the model eukaryote *S. cerevisiae*, the pleiotropic drug resistance protein 5 (Pdr5p) and the polyamine transporter 1 (Tpo1p), respectively, stand out as the transporters conferring resistance to a wider array of drugs and xenobiotics^9, 10^. Furthermore, both proteins confer specific resistance to agriculture-relevant stress agents. Tpo1p, for example, is a determinant of yeast resistance to auxinic herbicides such as 2,4-dichlorophenoxyacetic acid (2,4-D)^11^. Pdr1p, a transcription factor also determining 2,4-D resistance, is responsible for the activation of Tpo1p expression upon sudden exposure to the herbicide^11^. Tpo1p is also involved in the resistance to other chemical compounds, such as the herbicide barban or the fungicide mancozeb, and to toxic ions such as Cd^2+^ and Al^3+^ ^12^. Interestingly, the expression of an MFS member from Arabidopsis*, At*ZIFL1, in yeast also resulted in increased resistance to 2,4-D, and to the Tl^3+^ and Al^3+^ cations^12^. The *S. cerevisiae* Pdr5p transporter has also been reported to be both under Pdr1p transcriptional control upon exposure to 2,4-D and essential for the resistance to this herbicide^11^.

In the present study, we analysed the potential of the *S. cerevisiae* Tpo1p and Pdr5p transporters to confer multidrug resistance in *A. thaliana*. Transgenic plants expressing either of these transporters were generated and then challenged with diverse chemical species to which Tpo1p and/or Pdr5p mediate resistance in yeast. We found that heterologous expression of *ScTPO1* or *ScPDR5* in Arabidopsis resulted in enhanced plant tolerance to both the 2,4-D and barban herbicides and either to the fungicide mancozeb, the herbicides alachlor and metolachlor or to the fungicide cycloheximide, respectively. Moreover, resistance to toxic concentrations of several cations, namely Co^2+^, Cu^2+^, Ni^2+^, Al^3+^ and Cd^2+^, was observed in plants expressing *ScTPO1*. Thus, our results point to the yeast transporters Tpo1p and Pdr5p as important tools to improve plant performance in xenobiotic-contaminated environments, with Tpo1p showing to be particularly effective in mediating resistance against a broad range of toxic substances.

## Results

### Heterologous expression of the yeast Tpo1p or Pdr5p transporters does not affect normal development of Arabidopsis plants

Previous work showed that Tpo1p and Pdr5p are both involved in *S. cerevisiae* resistance to herbicides such as 2,4-D^11^. To evaluate whether the expression of these yeast transporters would also be able to confer plant xenobiotic resistance, we transformed *A. thaliana* plants with the coding sequences of either the *TPO1* or the *PDR5 S. cerevisiae* genes under the control of the strong constitutive 35S promoter.

**Figure 1:**
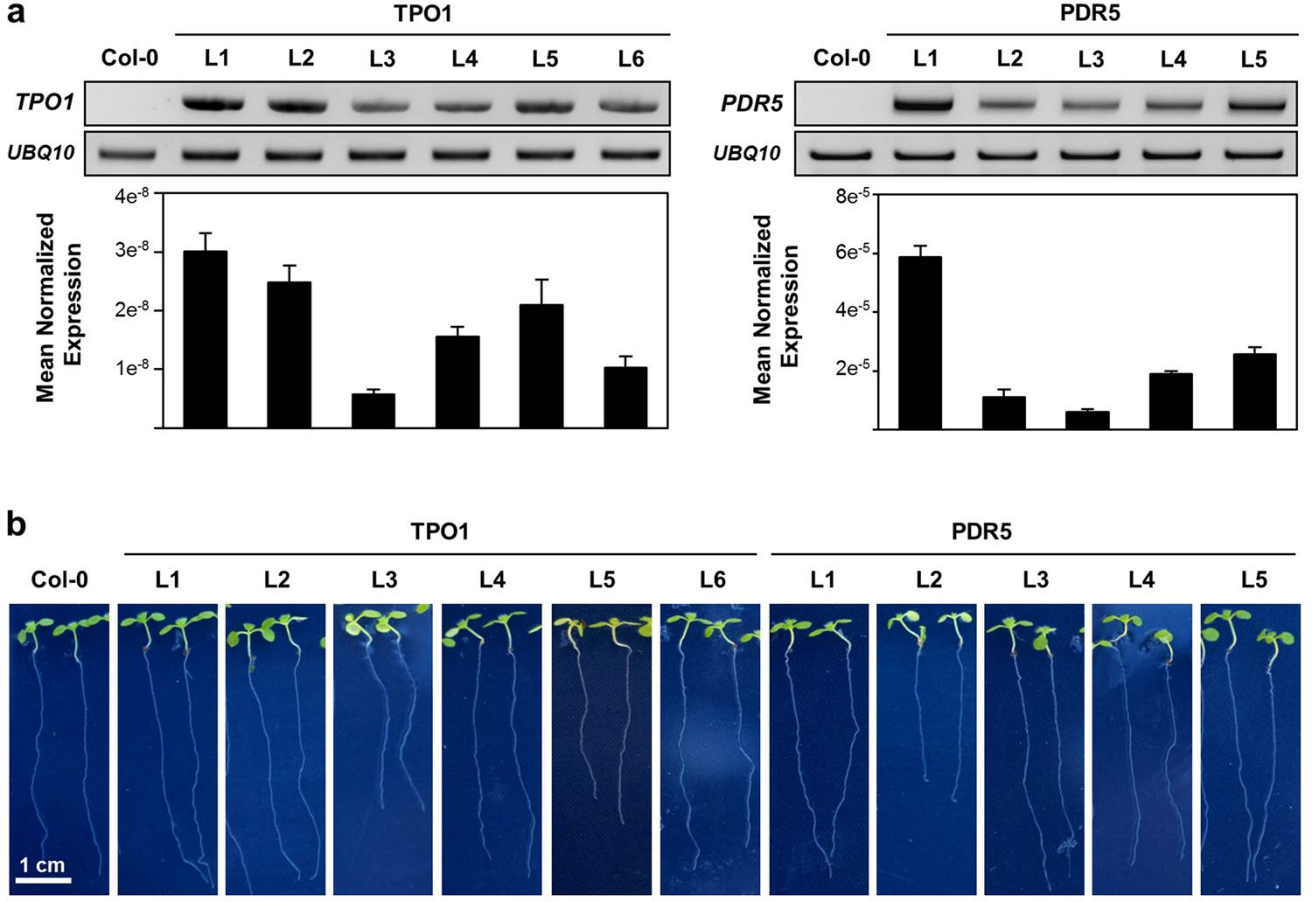
Transgene expression and phenotype under control conditions of transgenic Arabidopsis seedlings expressing the yeast Tpo1p or Pdr5p transporters. (**a**) Standard RT-PCR (upper panels) and real-time RT-PCR (lower panels) analysis of *S. cerevisiae TPO1* (left) and *PDR5* (right) transcript levels in 7-d old seedlings of the wild type (Col-0) and TPO1 (TPO1L1-L6) or PDR5 (PDR5L1-L5) transgenic lines. Expression of the *UBQ10* gene was used as a loading control or as a reference gene for standard RT-PCR and real-time RT-PCR, respectively. Values represent means ± SE (*n* = 4), with similar results being obtained in two independent experiments. (**b**) Representative images of 7-d old seedlings of the wild type (Col-0) and TPO1 (TPO1L1-L6) or PDR5 (PDR5L1-L5) transgenic lines grown under control conditions.

All isolated Arabidopsis transgenic lines expressed the corresponding transcript (Fig. 1a, upper panels), with real-time RT-PCR revealing levels about 1000-fold higher for the *ScPDR5* transgene than for *ScTPO1*, and still considerable differences in expression levels, though within the same order of magnitude, between lines expressing the same yeast transporter, possibly due to differences in transgene copy number (Fig. 1a, lower panels). In general, the independent transgenic lines obtained showed wild-type behaviour during development under control conditions (Fig. 1b and Table 1), although the TPO1 transgenic lines L3 and L5 and the PDR5 transgenic line L2 were found to exhibit delayed growth when compared with wild-type plants (Fig. 1b). Indeed, seedlings corresponding to these lines displayed slower elongation of the primary root (PR) than the wild type, but enhanced lateral root (LR) development, as measured through LR density and total length (Table 1). From a biotechnological point of view, the introduction of a transgene should confer (a) particular advantage(s) without negatively affecting other equally desirable traits and, for this reason, the TPO1 L3, TPO1 L5 and PDR5 L2 transgenic lines were discarded from further analyses.

**Table 1:** Growth parameters under control conditions of transgenic Arabidopsis lines expressing the yeast Tpo1p or Pdr5p transporters.

Genotypes	FW (mg per plant)	PRE (cm)	LRD (LR/cm)	TLRL (cm)
Col-0	25.59 ± 2.62	4.27 ± 0.48	1.47 ± 0.32	5.44 ± 1.15
*ScTPO1*-expressing				
TPO1L1	26.57 ± 3.38 (0.277571)	4.52 ± 0.30 (0.059858)	1.37 ± 0.32 (0.220766)	6.03 ± 1.30 (0.188536)
TPO1L2	27.33 ± 4.18 (0.183777)	4.16 ± 0.28 (0.232608)	1.62 ± 0.28 (0.099624)	5.43 ± 0.66 (0.493820)
TPO1L3	21.40 ± 5.12 (0.037274)	2.99 ± 0.62 (1.511e^−05^)	2.80 ± 0.99 (0.000142)	7.46 ± 2.13 (0.000142)
TPO1L4	24.73 ± 2.04 (0.253392)	4.13 ± 0.38 (0.202709)	1.52 ± 0.38 (0.350817)	5.72 ± 0.91 (0.308970)
TPO1L5	21.58 ± 3.69 (0.017224)	2.98 ± 0.82 (9.023e^−05^)	2.09 ± 0.66 (0.006238)	6.97 ± 2.23 (0.006238)
TPO1L6	26.98 ± 5.17 (0.268335)	4.33 ± 0.46 (0.373284)	1.48 ± 0.37 (0.455183)	5.12 ± 1.32 (0.455183)
*ScPDR5*-expressing				
PDR5L1	26.46 ± 3.97 (0.318385)	4.30 ± 0.46 (0.435980)	1.80 ± 0.76 (0.075609)	6.05 ± 0.98 (0.149458)
PDR5L2	22.26 ± 3.83 (0.039194)	2.89 ± 0.68 (1.230e^−05^)	2.56 ± 0.77 (0.000119)	9.88 ± 2.15 (0.000135)
PDR5L3	26.46 ± 4.57 (0.334319)	4.48 ± 0.24 (0.088359)	1.34 ± 0.69 (0.267862)	5.29 ± 1.29 (0.411617)
PDR5L4	26.38 ± 2.26 (0.279327)	4.17 ± 0.30 (0.271002)	1.58 ± 0.28 (0.162667)	5.60 ± 0.85 (0.382948)
PDR5L5	25.01 ± 4.78 (0.391651)	4.26 ± 0.42 (0.468568)	1.50 ± 0.11 (0.357897)	5.03 ± 0.64 (0.212272)

Shoot biomass (fresh weight, FW), primary root elongation (PRE), lateral root density (LRD) and total lateral root length (TLRL) of 7-d (PRE) or 14-d (FW, LRD, TLRL) old seedlings from the wild type (Col-0) or *ScTPO1*- and *ScPDR5*-expressing lines grown in control medium. Values represent means ± SD (*n = *8), with similar results being obtained in three independent experiments performed with different seed batches. Numbers in parentheses indicate the *P* value (comparison with the wild type) obtained by Student’s *t*-test.

### The yeast Tpo1p and Pdr5p transporters are also targeted to the plasma membrane in plant cells

**Figure 2:**
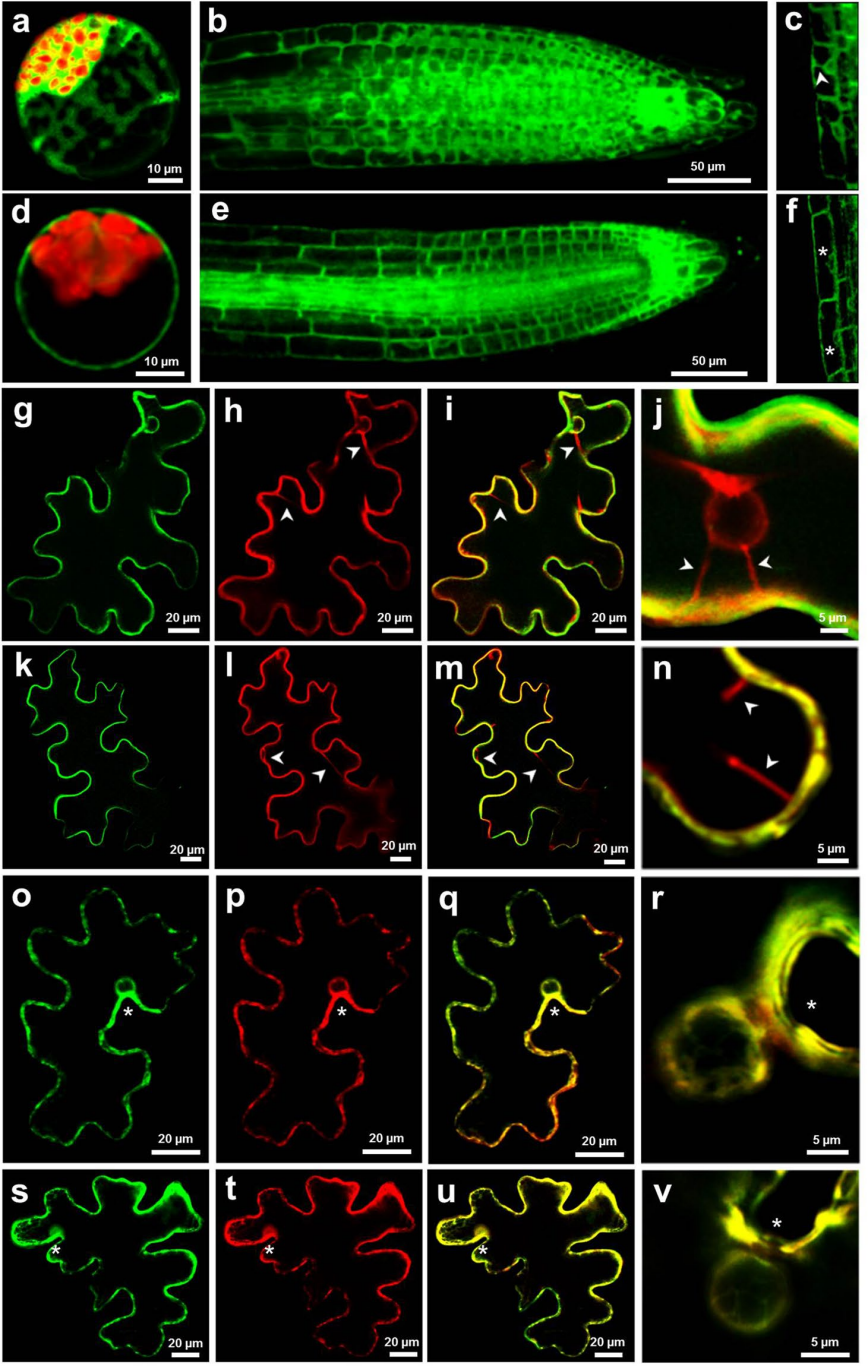
Subcellular localisation of the yeast Tpo1p and Pdr5p transporters in plant cells. (**a**–**f**) Confocal laser scanning microscopy images of Arabidopsis mesophyll protoplasts (**a**,**d**) or transgenic root tips (**b**,**c**,**e**,**f**), transiently or stably expressing either YFP alone (**a**,**b**,**c**) or the *ScTPO1-YFP* fusion (**d**,**e**,**f**), respectively, under the control of the 35S promoter. Root epidermal cell close-ups (**c**,**f**) are shown. The YFP and chloroplast autofluorescence signals are visualized by green and red coloration, respectively. (**g–v**) Confocal laser scanning microscopy images of individual tobacco leaf epidermal cells transiently expressing the *ScTPO1-GFP* (**g**,**i**,**j**,**o**,**q**,**r**) or *ScPDR5-GFP* (**k**,**m**,**n**,**s**,**u**,**v**) fusions and/or the tonoplast marker Ɣ-TIPmCherry (**h**,**i**,**j**,**l**,**m**,**n**) or the plasma-membrane marker PIP2A-mCherry (**p**,**q**,**r**,**t**,**u**,**v**) under the control of the 35S promoter. Merged images of whole-cell views (**i**,**m**,**q**,**u**) or nucleus close-ups (**j**,**n**,**r**,**v**) are shown. Arrowheads point to transvacuolar strands, and asterisks indicate fluorescence signals approaching the nucleus only on the side facing the exterior of the cell (**o**–**v**) or surrounding the nucleus (**f**). The GFP and mCherry signals are visualized by green and red coloration, respectively.

We next analysed the subcellular localisation of the Tpo1p and Pdr5p transporters in plant cells by means of translational fusions with yellow fluorescent protein (YFP) or green fluorescent protein (GFP) (Fig. 2). Both protoplasts isolated from Arabidopsis leaf mesophyll cells and whole Arabidopsis plants were transformed with either the transporter-YFP fusion or YFP alone driven by the 35S promoter. In parallel, *Nicotiana tabacum* leaves were co-transfected with either the *35S:ScTPO1-GFP* or *35S:ScPDR5-GFP* constructs and a plasma-membrane or a tonoplast marker.

As shown in Fig. 2a–f, upon both transient expression in isolated protoplasts and stable expression in transgenic plants, Tpo1p localised to the plasma membrane of Arabidopsis cells. Accordingly, in *N. tabacum* leaves, the Tpo1p-GFP fusion co-localised with the plasma-membrane marker (Fig. 2o–r) but not with the tonoplast marker (Fig. 2g–j), confirming that, *in planta* like in yeast, the *S. cerevisiae* Tpo1p transporter localises to the plasma membrane.

Although the fluorescence signal arising from the *ScPDR5-YFP* construct was not detectable in Arabidopsis cells, co-localisation of the Pdr5p-GFP fusion with the plasma-membrane (Fig. 2s–v) but not with the tonoplast (Fig. 2k–n) marker was again observed in *N. tabacum* leaves, indicating that the yeast Pdr5p transporter also maintains its subcellular localisation when heterologously expressed in plant cells.

### The yeast Tpo1p and Pdr5p transporters confer resistance to the 2,4-D herbicide in Arabidopsis

**Figure 3:**
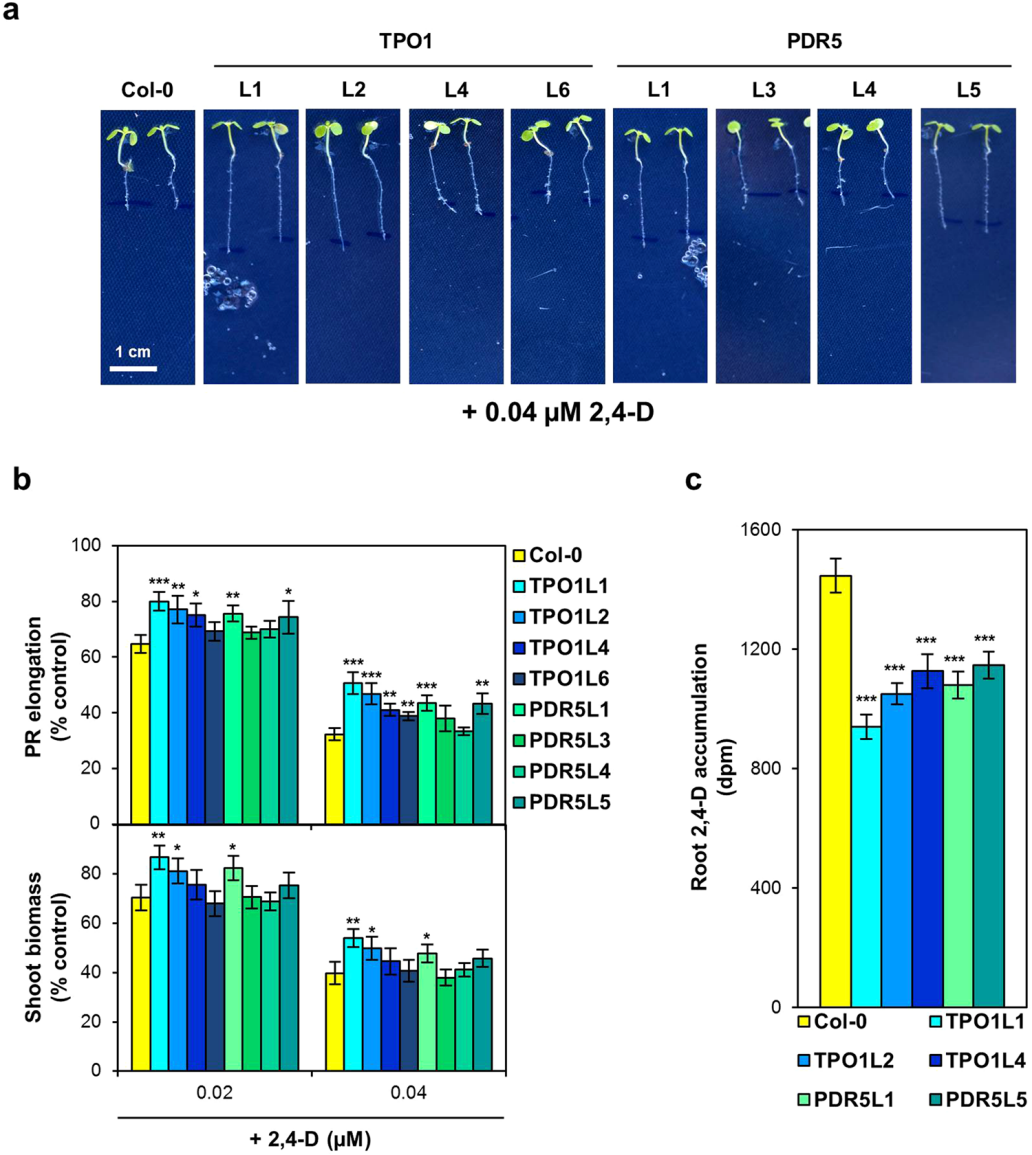
Effects of a 2,4-D challenge on transgenic Arabidopsis lines expressing the yeast Tpo1p or Pdr5p transporters. (**a**) Representative images of 7-d old seedlings of the wild type (Col-0) and TPO1 (TPO1L1, L2, L4, L6) or PDR5 (PDR5L1, L3, L4, L5) transgenic lines grown on medium containing 2,4-D. Plant growth under control conditions is shown in Fig. 1 and Table 1. (**b**) Effect of 2,4-D toxicity on PR elongation (upper panel) and shoot biomass (lower panel) of seedlings of the wild type (Col-0) and TPO1 (TPO1L1, L2, L4, L6) or PDR5 (PDR5L1, L3, L4, L5) transgenic lines. (**c**) Accumulation of ^14^C-2,4-D in root tips from 5-d old seedlings of the wild type (Col-0) and TPO1 (TPO1L1, L2, L4) or PDR5 (PDR5L1, L5) transgenic lines. Values represent means ± SD (*n* = 8), with similar results being obtained in three independent experiments performed with different seed batches. Asterisks denote statistically significant differences between *ScTPO1*- or *ScPDR5*-expressing lines and the wild type (*P < 0.05, **P < 0.01, ***P < 0.001; Student’s *t*-test).

In *S. cerevisiae*, both the Tpo1p and the Pdr5p transporters are known to be involved in the resistance to chlorinated phenoxyacetic acid herbicides such as 2,4-D^11^. To investigate whether heterologous expression of the yeast *ScTPO1* or *ScPDR5* genes can confer plant tolerance to these compounds, we analysed the response of the Arabidopsis *ScTPO1*- and *ScPDR5*-expressing lines to a 2,4-D challenge (Fig. 3).

At the level of PR elongation, the TPO1 transgenic lines L1, L2 and L4 and the PDR5 transgenic lines L1 and L5 were clearly less affected by the inhibitory effects of the herbicide than the wild type and the other transgenic lines (Fig. 3a and b, upper panel). The increase in plant tolerance ranged from 15 to 23% after a 0.02 µM 2,4-D challenge and was even more pronounced at 0.04 µM 2,4-D, with an increase between 20 and 57%. Furthermore, a higher shoot biomass was detected in the *ScTPO1*-expressing lines L1 and L2 and in the *ScPDR5*-expressing line L1 (Fig. 3b, lower panel), with a rise in tolerance very similar to that observed for the roots (15–23% at the lowest and 20–36% at the highest concentration of the herbicide). Importantly, the magnitude of the 2,4-D response largely correlated with the levels of heterologous expression of the corresponding yeast transporter in each transgenic line (see Fig. 1a). Despite the increased resistance to this auxinic herbicide, our results indicate that heterologous expression of *ScTPO1* and *ScPDR5* in *Arabidopsis* does not affect endogenous auxin metabolism, as PR elongation in transgenic plants treated with the native auxin indole-acetic acid (IAA) was similar to that of wild-type plants (Supplementary Fig. S1).

The mechanism by which Tpo1p confers 2,4-D resistance in yeast is the extrusion of this compound from cells^12^. Consistent with this, our measurements of ^14^C-2,4-D accumulation in roots were lower for the three TPO1 transgenic lines exhibiting 2,4-D resistance at the level of PR elongation than for wild-type plants (Fig. 3c). The two PDR5 lines whose PRs displayed reduced 2,4-D sensitivity also accumulated significantly less herbicide than the wild type (Fig. 3c), suggesting that 2,4-D resistance conferred by Pdr5p involves a mechanism similar to that proposed for Tpo1p.

### Tpo1p and Pdr5p confer tolerance to several other agricultural pesticides in yeast and Arabidopsis

**Figure 4:**
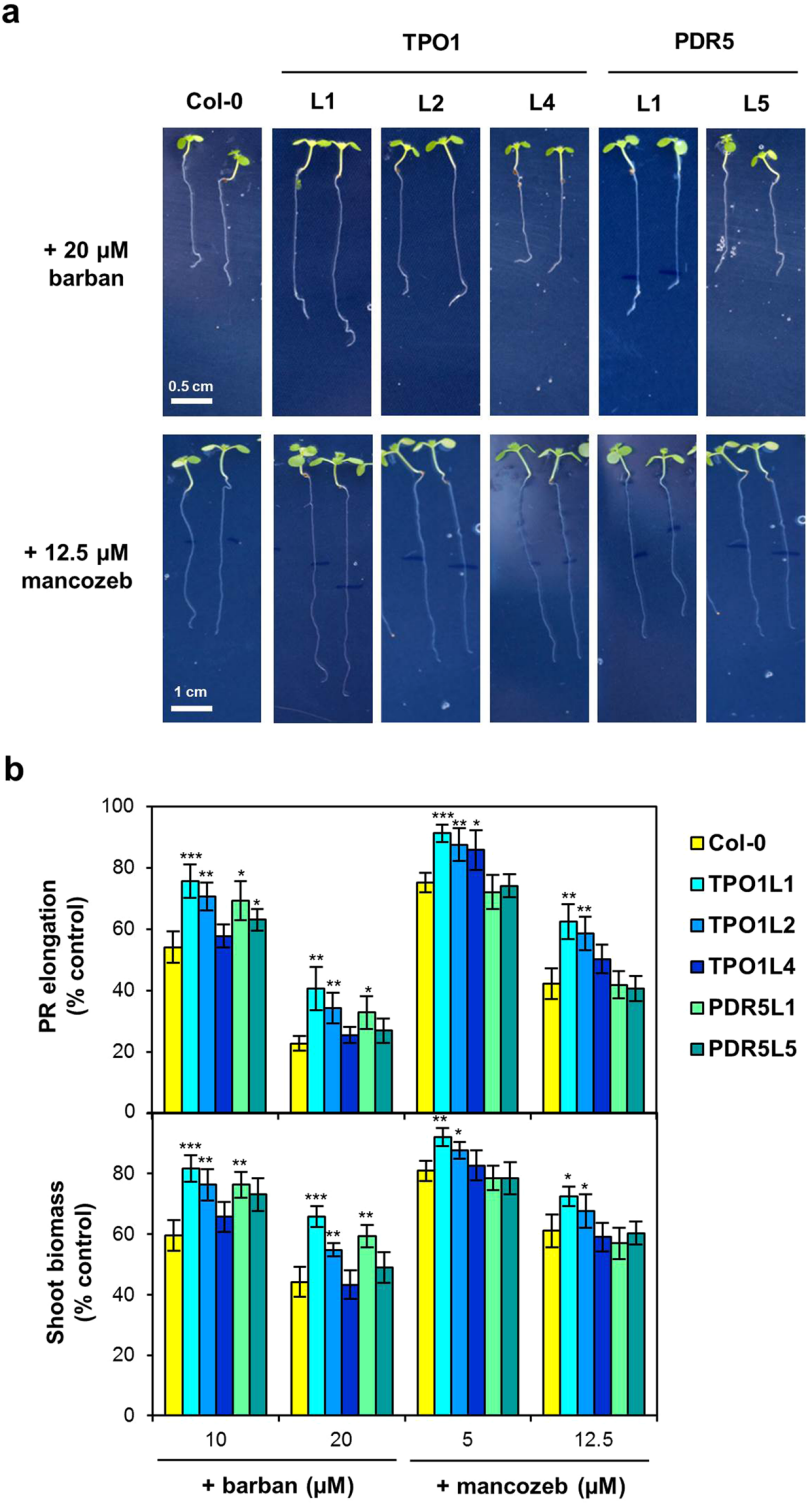
Effects of a barban or mancozeb challenge on transgenic Arabidopsis lines expressing the yeast Tpo1p or Pdr5p transporters. (**a**) Representative images of 7-d old seedlings of the wild type (Col-0) and TPO1 (TPO1L1, L2, L4) or PDR5 (PDR5L1, L5) transgenic lines grown on media containing barban (upper panel) or mancozeb (lower panel). Plant growth under control conditions is shown in Fig. 1 and Table 1. (**b**) Effect of barban or mancozeb toxicity on PR elongation (upper panel) and shoot biomass (lower panel) of seedlings of the wild type (Col-0) and TPO1 (TPO1L1, L2, L4) or PDR5 (PDR5L1, L5) transgenic lines. Values represent means ± SD (*n* = 8), with similar results being obtained in three independent experiments performed with different seed batches. Asterisks denote statistically significant differences between *ScTPO1*- or *ScPDR5*-expressing lines and the wild type (*P < 0.05, **P < 0.01, ***P < 0.001; Student’s *t*-test).

In addition to 2,4-D, the yeast Δ*tpo1* mutant displays decreased resistance to other compounds of agricultural interest, namely barban, a herbicide, and mancozeb, a fungicide^12^, both of which inhibit the growth of Arabidopsis plants (Supplementary Fig. S2a). To examine the possibility that the Tpo1p and Pdr5p transporters confer resistance to these pesticides also in plants, we challenged the Arabidopsis *ScPDR5* and *ScTPO1*-expressing lines displaying the highest resistance to 2,4-D with either barban or mancozeb. With the exception of TPO1L4, PR elongation in the transgenic lines was less inhibited by barban than in the wild type, with increases in tolerance ranging between 17% and 40% or 44% and 79% for the lowest or highest barban concentration, respectively, although at the highest concentration tested the PDR5L5 line also behaved similarly to wild-type plants (Fig. 4). The fresh weight of transgenic shoots was also higher than that of wild-type shoots, showing increases between 24% and 49%, except for TPO1L4 and PDR5L5 (Fig. 4b, lower panel). As for mancozeb, the growth of *ScTPO1*-expressing plants was less sensitive to the effects of the fungicide than that of the *ScPDR5* transgenic lines or wild-type plants (Fig. 4) although for TPO1L4, the plant line expressing lower amounts of *ScTPO1* transcript (see Fig. 1a), no alterations in mancozeb sensitivity were observed at the highest concentration (Fig. 4b). Although in general *ScTPO1* transgenic plants resisted better to barban than to mancozeb, the root and shoot tolerance to the latter compound still increased by 17–48% and 8–19%, respectively. These results prompted us to investigate the response of the yeast Δ*pdr5* mutant to barban and mancozeb. In line with what was observed *in planta*, yeast cells lacking Pdr5p were more sensitive to barban than the wild-type, whereas the wild-type and the mutant strain were equally sensitive to mancozeb (Supplementary Fig. S3). In the mancozeb experiment, we included the Δ*tpo1* strain as a control, given that this mutant has been previously reported to display hypersensitivity to the fungicide^12^.

**Figure 5:**
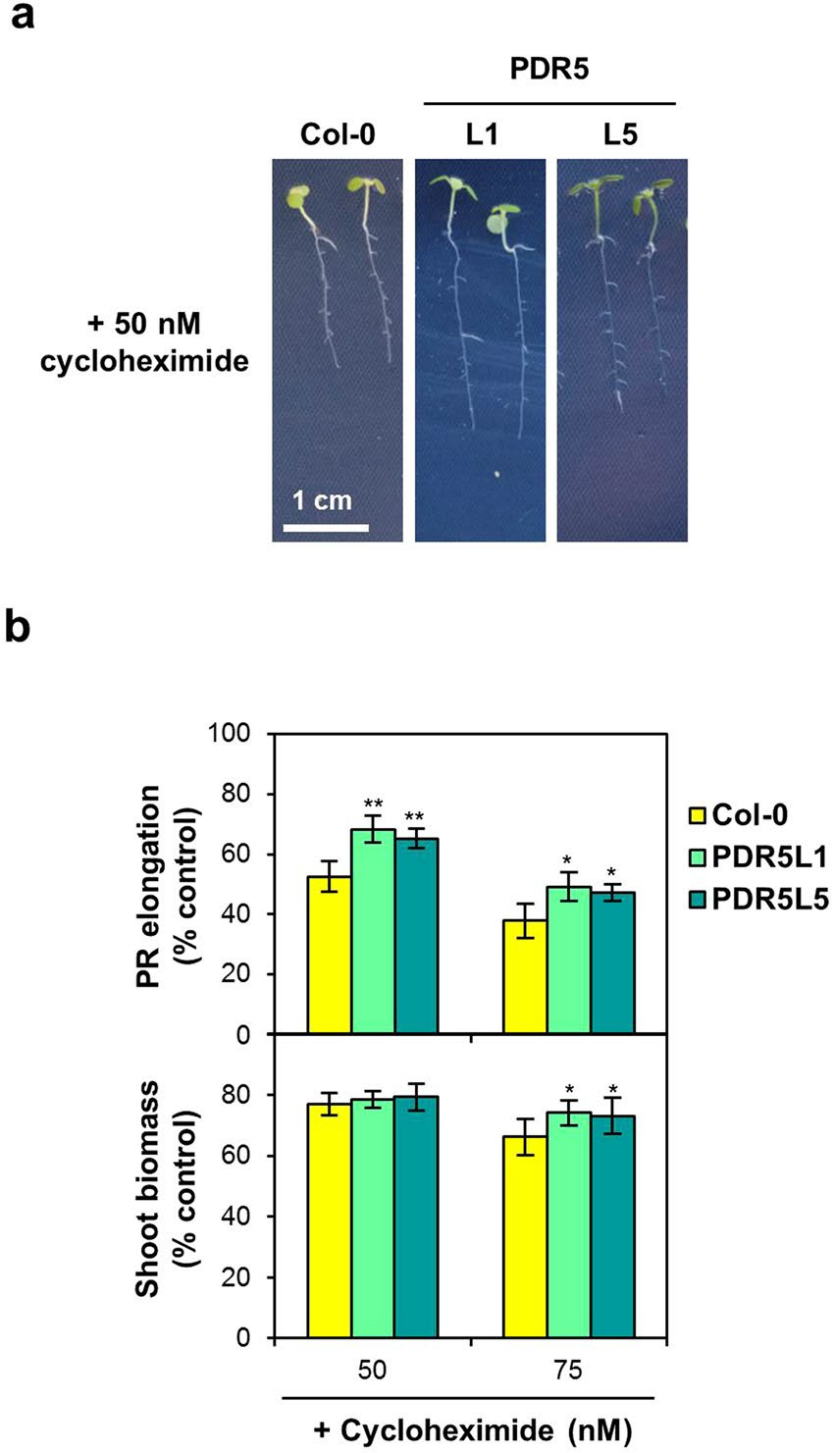
Effects of a cycloheximide challenge on transgenic Arabidopsis lines expressing the yeast Pdr5p transporter. (**a**) Representative images of 7-d old seedlings of the wild type (Col-0) and PDR5 (PDR5L1, L5) transgenic lines grown on medium containing cycloheximide. Plant growth under control conditions is shown in Fig. 1 and Table 1. (**b**) Effect of cycloheximide toxicity on PR elongation (upper panel) and shoot biomass (lower panel) of seedlings of the wild type (Col-0) and PDR5 (PDR5L1, L5) transgenic lines. Values represent means ± SD (*n* = 8), with similar results being obtained in three independent experiments performed with different seed batches. Asterisks denote statistically significant differences between *ScPDR5*-expressing lines and the wild type (*P < 0.05, **P < 0.01; Student’s *t*-test).

Encouraged by our finding that the multidrug resistance conferred by two yeast plasma-membrane transporters also functions in Arabidopsis, and owing to the decreased resistance of *S. cerevisiae PDR5* mutants to cycloheximide^13^, we next analysed the effects of Pdr5p expression on plant responses to this compound, which has been used in agriculture as a large-spectrum fungicide under the name actidione. Both the PDR5L1 and PDR5L5 transgenic lines were tested at two different concentrations of cycloheximide, exhibiting a longer PR (~27% increase) and higher fresh shoot weight than wild-type plants (Fig. 5). The effect on shoot weight was subtle (~11% increase) and only felt at the highest concentration tested (Fig. 5b, lower panel), most likely because this parameter is less affected by cycloheximide than elongation of the PR (Supplementary Fig. S2b).

**Figure 6:**
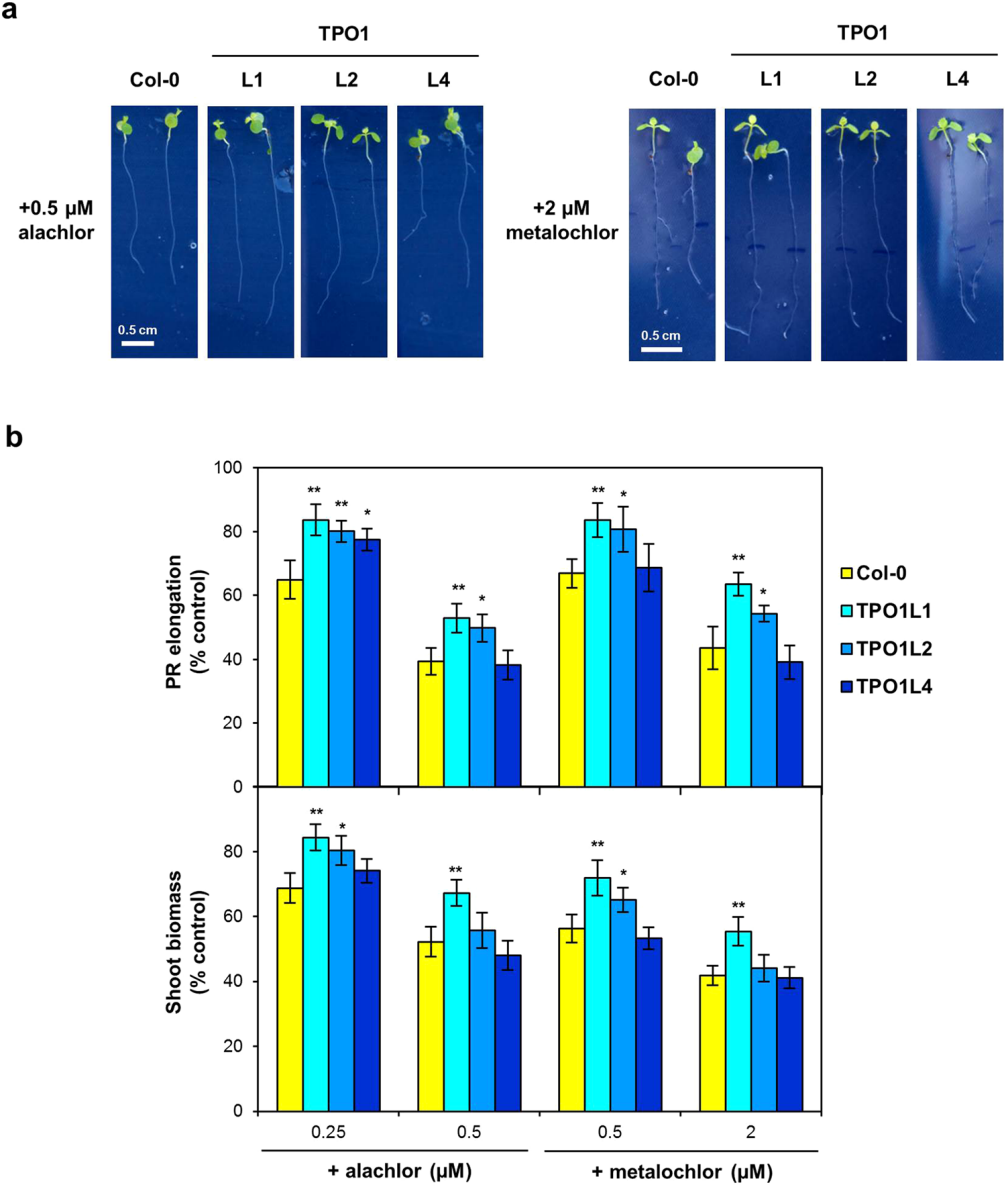
Effects of an alachlor or metalochlor challenge on transgenic Arabidopsis lines expressing the yeast Tpo1p transporter. (**a**) Representative images of 7-d old seedlings of the wild type (Col-0) and TPO1 (TPO1L1, L2, L4) transgenic lines grown on media containing alachlor (left panel) or metalochlor (right panel). Plant growth under control conditions is shown in Fig. 1 and Table 1. (**b**) Effect of alachlor or metalochlor toxicity on PR elongation (upper panel) and shoot biomass (lower panel) of seedlings of the wild type (Col-0) and TPO1 (TPO1L1, L2, L4) transgenic lines. Values represent means ± SD (*n* = 8), with similar results being obtained in three independent experiments performed with different seed batches. Asterisks denote statistically significant differences between *ScTPO1*-expressing lines and the wild type (*P < 0.05, **P < 0.01; Student’s *t*-test).

We also investigated the *in planta* role of Tpo1p in the resistance to other pesticides not yet reported in yeast, such as alachlor and metolachlor. Both these herbicides markedly impaired Arabidopsis growth even at very low concentrations (Supplementary Fig. S2c). As seen in Fig. 6, both PR elongation and shoot biomass of two independent plant lines expressing *ScTPO1* (TPO1L1 and TPO1L2) were less affected by alachlor and metolachlor than wild-type plants, although at the higher concentrations tested TPO1L2 shoot biomass was similar to that of the wild type (Fig. 6b, lower panel). The *ScTPO1* transgenic lines exhibited a moderate increase in the tolerance to both herbicides, ranging from 23 to 34% or 17 to 29% and 21 to 46% or 16 to 33% for alachlor and metolachlor at the PR elongation or shoot biomass levels, respectively. Tpo1p-mediated resistance to both alachlor and metolachlor was also observed in yeast, with the Δ*tpo1* mutant strain showing enhanced sensitivity to these compounds relative to the wild type when grown in either solid (Supplementary Fig. S4a) or liquid (Supplementary Fig. S4b) media.

### Tpo1p confers plant tolerance to rhizotoxic cations

Tpo1p has been previously reported to be involved in the resistance to some metal cations, such as Al^3+^ or Cd^2+^, in yeast^12^. In plants, transition metals like Co^2+^, Cu^2+^ or Ni^2+^ act as micronutrients, but become toxic at high concentrations.

**Figure 7:**
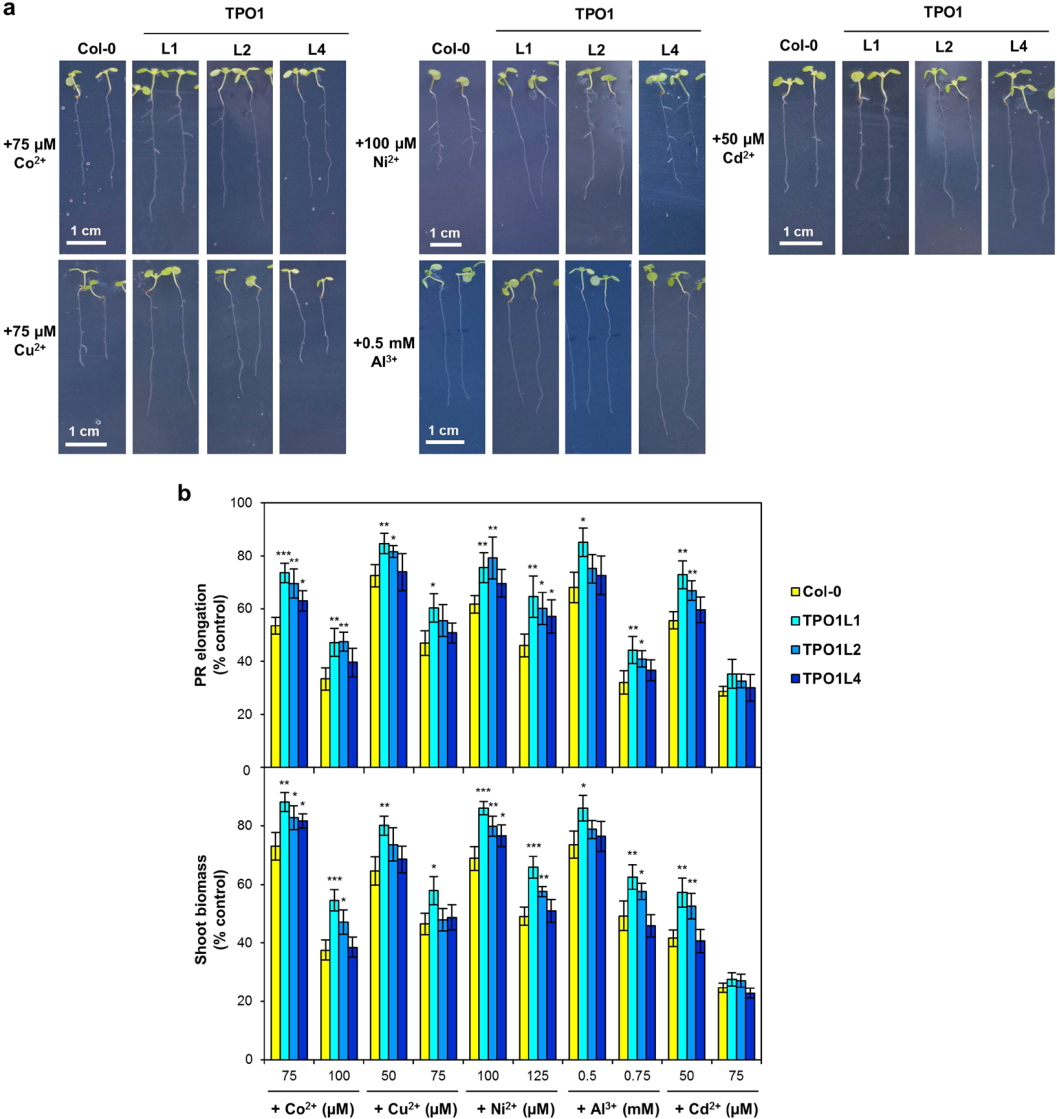
Effects of rhizotoxic ion challenge on transgenic Arabidopsis lines expressing the yeast Tpo1p transporter. (**a**) Representative images of 7-d old seedlings of the wild type (Col-0) and TPO1 (TPO1L1, L2, L4) transgenic lines grown on media containing Co^2+^, Cu^2+^, Ni^2+^, Al^3+^ or Cd^2+^. Plant growth under control conditions is shown in Fig. 1 and Table 1. (**b**) Effect of Co^2+^, Cu^2+^, Ni^2+^, Al^3+^ or Cd^2+^ toxicity on PR elongation (upper panel) and shoot biomass (lower panel) of seedlings of the wild type (Col-0) and TPO1 (TPO1L1, L2, L4) transgenic lines. Values represent means ± SD (*n* = 8), with similar results being obtained in three independent experiments performed with different seed batches. Asterisks denote statistically significant differences between *ScTPO1*-expressing lines and the wild type (*P < 0.05, **P < 0.01, ***P < 0.001; Student’s *t*-test).

To investigate the ability of the yeast Tpo1p transporter to confer resistance to metal cations in plants, we analysed the growth of Arabidopsis *ScTPO1*-expressing lines upon exposure to two different concentrations of the Co^2+^, Cu^2+^, Ni^2+^, Al^3+^ or Cd^2+^ ions, selected based on our previously reported physiological analyses^14^. From Fig. 7 it is clear that transgenic lines TPO1L1 and TPO1L2 exhibited a longer PR than wild-type plants when grown in the presence of at least one concentration of each of the five cations, while TPO1L4 showed enhanced resistance only when treated with the lowest concentration of Co^2+^ or the highest concentration of Ni^2+^ (Fig. 7a and b, upper panel). The rise in plant tolerance was between 42%, at the maximum Co^2+^ concentration, and 13%, at the lowest Cu^2+^ concentration. Similar results were observed when fresh shoot weight was measured (Fig. 7b, lower panel), with tolerance to the cations being enhanced 11–45%, although in the Cu^2+^ treatments only TPO1L1 showed to be more resistant than the wild type. None of the TPO1 transgenic lines displayed a phenotype when challenged with the highest concentration of Cd^2+^, probably because this level of toxicity is high enough to surpass Tpo1p-mediated resistance.

We also checked whether Al^3+^, Cd^2+^, Co^2+^, Cu^2+^ and Ni^2+^ accumulation would be affected in *ScTPO1*-expressing plants upon challenge with each of these cations by comparing their respective content in transgenic and wild-type seedlings. Overall, no significant differences were observed between the plant line displaying the strongest transgene expression, TPO1L1, and the wild type, except for Al^3+^-treated plants, where the content of this cation was found to be higher in the transgenic line (Table 2).

**Table 2:** Elemental analysis of a transgenic Arabidopsis line expressing the yeast Tpo1p transporter.

	Col-0	TPO1L1
Co^2+^	298.36 ± 43.78	306.48 ± 22.42 (0.742)
Cu^2+^	77.84 ± 11.11	81.42 ± 22.16 (0.782)
Ni^2+^	186.38 ± 19.78	192.97 ± 94.67 (0.896)
Cd^2+^	311.36 ± 15.16	285.70 ± 63.03 (0.459)
Al^3+^	148.30 ± 20.89	181.44 ± 13.34 (0.037)

Total cation concentration (µg g^−1^ DW) of wild-type and transgenic *ScTPO1*-expressing Arabidopsis plants challenged with 0.5 mM Al^3+^, 50 µM Cd^2+^, 75 µM Co^2+^, 50 µM Cu^2+^ and 100 µM Ni^2+^ (means ± SD, *n* = 4). Numbers in parentheses indicate the *P* value (comparison with the wild type) obtained by Student’s *t*-test.

Despite the increased tolerance to all five examined cations conferred by heterologous expression of the yeast Tpo1p transporter in Arabidopsis, and in contrast to what has been reported for Al^3+^ and Cd^2+^, we were unable to detect a differential response between the yeast Δ*tpo1* mutant and the wild-type strain to the transition metals Co^2+^, Cu^2+^ or Ni^2+^ (Supplementary Fig. S5).

## Discussion

Soil contamination is a major hindrance to plant growth and productivity, and hence the development of effective strategies to overcome the toxic effects of soil pollutants is widely recognized as crucial. Here, we report the effects of transgenic expression of two *S. cerevisiae* plasma-membrane transporters, Tpo1p, belonging to the MFS, and Pdr5p, an ABC superfamily member, on tolerance of the model plant *A. thaliana* towards several toxic substances, including agricultural pesticides and heavy metals.

Under control conditions, heterologous expression of the yeast *ScTPO1* and *ScPDR5* genes in Arabidopsis did not affect the growth of the transgenic lines generated, with the exception of two *ScTPO1*- and one *ScPDR5*-expressing lines. In fact, transgene expression levels in these three lines were comparable to those in the remaining transgenic plants, suggesting that the observed delay in growth is not related to heterologous expression of the yeast transporters, but rather to the regions of the Arabidopsis genome disrupted by random insertion of the transgenes. Interestingly, expression of *ScPDR5* was always about three orders of magnitude higher than that of *ScTPO1*, despite the fact that both genes are being driven *in planta* by the same strong constitutive promoter, 35S. This discrepancy could be due to differential mRNA processing or stability and/or regulation of the transgenes at the transcription/translation levels^15^.

Globally, heterologous expression of the Tpo1p and Pdr5p yeast transporters in Arabidopsis conferred a moderate though significant increase in plant tolerance to a range of compounds. Generally, it might well be preferable for the plant to exhibit moderate resistance to several agents than to greatly resist a few. Still, enhancing the intensity of the observed broad tolerance could be highly desirable. One way to improve the transgenes’ efficiency would be to target them to a specific site in the plant genome, thus selecting the most convenient location for the insertion and avoiding collateral effects, such as the disruption of genes crucial to plant survival. Endonuclease-based technologies such as Clustered Regularly Interspaced Short Palindromic Repeats (CRISPR)-Associated (Cas)^16^, Zinc-Finger Nucleases (ZFNs)^17^ or Transcription Activator-Like Effector Nucleases (TALENs)^18^ may be used for this purpose. Also, depending on the plant species in which the transgene is inserted, other promoters that are either more efficient or whose activity is specific to a tissue or a developmental stage could be employed when desirable.

In yeast, the *ScTPO1* and *ScPDR5* transporter genes are activated by Pdr1p, a transcriptional regulator determining yeast resistance to two widely used herbicides, 2-methyl-4-chlorophenoxyacetic acid (MCPA) and 2,4-D^11^. In addition, Tpo1p has been shown to confer yeast resistance to the herbicide barban, the plant hormone IAA, the fungicide mancozeb, and the metal cations Cd^2+^ and Al^3+^ ^12^, whereas Pdr5p has been implicated in the resistance to a broad range of substances such as mycotoxins^19^, steroid hormones^20^, cycloheximide^21^, and a vast number of antifungal compounds^22^. Two possible explanations for the pleiotropic resistance promoted by Tpo1p and Pdr5p can be envisaged. On the one hand, both these transporters appear to be important for cellular processes other than multidrug resistance, and therefore resistance to xenobiotics could represent a collateral effect of the transporter’s function. For instance, Tpo1p was postulated to mediate cell extrusion of polyamines to regulate their intracellular concentration^23, 24^. Polyamines are small aliphatic cations critical to growth and development of both eukaryotes and prokaryotes^25^, and hence their regulation is pivotal to cell fitness. Also, Pdr5p is involved in ATP-dependent phospholipid translocation^26^ and could thus modulate the permeability of the plasma membrane by altering its lipid composition. On the other hand, at least in some instances, multidrug resistance may be conferred directly by Tpo1 and Pdr5. Indeed, many of the substances these transporters mediate resistance to are weak acid compounds, and it could well be that their dissociated counter-ions are Tpo1p and/or Pdr5p substrates^27^.

*ScTPO1*-expressing plants challenged with cations accumulated levels of Cd^2+^, Co^2+^, Cu^2+^ and Ni^2+^ similar to the wild type and higher levels of Al^3+^, which would suggest that the Tpo1p-mediated resistance mechanism does not involve extrusion of the cations from plant tissues. However, a direct mechanism of resistance cannot be excluded as the Tpo1p transporter could act at the level of organ partitioning of these cations, reducing the magnitude of their negative effects on plant growth through their plant repartition. The fact that the transgenic plants accumulated more Al^3+^ than the wild type but were still less affected by this cation, whose first symptom of toxicity is inhibition of root growth^28^, could support this hypothesis. Taken together, the findings that Tpo1p and Pdr5p are targeted to the same cellular membrane in yeast and transgenic Arabidopsis lines, that the biological processes in which these transporters have been implicated are conserved between plants and yeast, and that the resistance phenotypes conferred in transgenic plants are often reciprocal to those displayed by yeast loss-of-function mutants, strongly suggest that the general mechanisms by which xenobiotic resistance is achieved in Arabidopsis are the same as in yeast.

The weak acid 2,4-D is one of the ten most commonly used active pesticide ingredients in the U.S.^29^, and it is also extensively employed in agriculture worldwide. The importance of developing crops resistant to herbicidal compounds lies on the potential harmful effects of these substances on the crops they are protecting from weeds. Although varieties of some crops, such as cotton, corn and soybean, displaying resistance to 2,4-D (as well as to the glyphosate and glufosinate herbicides) have been developed and recently launched into the market^30^, weed resistance remains a major concern that could be alleviated by the broadening of the range of agricultural compounds crops are resistant to. In addition, the broad resistance achieved here with plants expressing the yeast Tpo1p transporter could allow farming in soils contaminated with heavy metals without altering the quality of the edible parts of crops, as the expression of this transporter did not lead to over-accumulation of toxic cations.

Homologs of the yeast Pdr5p in plants have proven pivotal in a wide range of processes such as xenobiotic detoxification, biotic stress resistance and phytohormone transport^31^. For example, *At*PDR9 was shown to transport auxinic compounds such as the herbicide 2,4-D and the endogenous auxin precursor indole-3-butyric acid (IBA)^32, 33^. *At*PDR8 has also been shown to be involved in IBA transport as well as to play roles in the resistance to ions such as Cd^2+^ and Na^+^, and contribute to plant defence from pathogens^34–38^. *At*PDR12, on the other hand, is involved in Pb^2+^ detoxification, resistance to the diterpenoid sclareol, and the uptake of the plant hormone abscisic acid (ABA)^39–41^. Although the involvement of MFS transporters in xenobiotic resistance has not been widely studied, and none of those closest to Tpo1p in Arabidopsis has been characterized, there are reported examples of the importance of the MFS in plant resistance to a range of insults. For instance, *At*ZIF1 and *At*ZIF2 are both involved in the tolerance to the Zn^2+^ cation^42, 43^, *At*ZIFL1 confers resistance to 2,4-D and to auxin^44^, and *At*ZIFL2 is important in maintaining Cs^2+^ and K^+^ homeostasis in Arabidopsis^45^. Finally, we show here that expression of the *S. cerevisiae* Tpo1p and Pdr5p in Arabidopsis enlarges the plant toolkit to successfully endure the effects of adverse soil conditions.

The present work demonstrates that the introduction of a single gene can expand the plant resistance repertoire from 2,4-D to not only other herbicides such as barban and the closely-related alachlor and metolachlor, but also to other active ingredients like the fungicides mancozeb and actidione (cycloheximide) and to soil contaminants such as metal ions. To our knowledge, the effects of barban and mancozeb on Arabidopsis were explored here for the first time. The results reported in this study could serve as the basis for future biotechnological strategies to improve plant performance in an increasingly challenging environment.

## Methods

### Plant materials and growth conditions

The *Arabidopsis thaliana* (L.) Heynh., ecotype Columbia (Col-0), was used as the wild type in all experiments. Plant transformation was achieved by the floral-dip method^46^ using *Agrobacterium tumefaciens* strain EHA105. Seeds were surface-sterilized and sown on Murashige and Skoog^47^ medium solidified with 0.8% agar, stratified for 3 d, placed in a growth chamber and transferred to soil after 2–3 weeks. Plants were cultivated under long-day conditions (16-h light, 22 °C/8-h dark, 18 °C) at 60% RH.

### Generation of transgenic lines

To generate *ScTPO1* (YLL028W) and *ScPDR5* (YOR153W) expression constructs, each full coding sequence, from start to stop codon, was PCR-amplified (Supplementary Table S1) using yeast genomic DNA. The resulting 1761-bp and 4536-bp fragments, corresponding to the *ScTPO1* and *ScPDR5* genes, respectively, were independently inserted via the *Xho*I/SpeI restriction sites into the pBA002 plasmid. After agroinfiltration of the resulting constructs into wild-type plants, six and five independent transformants showing significant expression of *ScTPO1* and *ScPDR5*, respectively, were recovered.

### Gene expression analyses

Standard RT-PCR analyses were conducted as previously described^48^ using primers designed to detect *ScTPO1, ScPDR5*, and *UBQ10* (*UBIQUITIN10*) expression (Supplementary Table S1). The results shown are representative of three independent experiments. Real-time RT-PCR was performed using specific primers (Supplementary Table S1) on the CFX384 Touch Real-Time PCR Detection System (Bio-Rad) using the Luminaris mix (Thermoscientific) according to the manufacturer’s instructions. For each condition tested, two RNA extractions from different biological samples and two reverse transcription reactions for each biological repeat were performed. Data were processed using Q-Gene^49^ that took the respective primer efficiency into consideration.

### Subcellular localisation studies

To generate Tpo1p and Pdr5p protein fusions with YFP and GFP, each coding sequence, excluding the stop codon, was PCR-amplified (Supplementary Table S1) using yeast genomic DNA as a template and independently inserted under the control of the 35S promoter via the *XhoI/PacI* restriction sites into the YFP- or GFP-tagged versions of the pBA002 plasmid. Ten stable transgenic lines displaying a strong fluorescence signal in the root were recovered upon transformation of wild-type plants with the Pro*35S:ScTPO1-YFP* construct, whereas no fluorescence signal was detected in the two isolated stable transgenic lines expressing significant expression of the *ScPDR5-YFP* fusion. *Arabidopsis* protoplasts were generated as previously described^50^ and transfected with the YFP constructs by polyethylene glycol (PEG) transformation^51^. Transient coexpression of the GFP constructs with the tonoplast marker γ-Tonoplast Intrinsic Protein (TIP)-mCherry or the plasma-membrane marker Plasma membrane Intrinsic Protein 2A (PIP2A)-mCherry^52^ and the pBIN-NA construct^53^ in leaf abaxial epidermal cells of *Nicotiana tabacum* was performed via agroinfiltration using *A. tumefaciens* strain GV3101.

### Yeast manipulations

The parental *Saccharomyces cerevisiae* strain BY4741 and the derived deletion mutants BY4741_*Δtpo1* and BY4741_*Δpdr5* previously described^11^ were used. Growth curve and spot assays were conducted in minimal growth medium MMB liquid or agarized^12^ and supplemented with the indicated compound (Sigma-Aldrich) at the desired concentration. Results presented are representative of three independent experiments.

### Plant phenotypical assays

Root assays were performed as described by Remy and Duque^14^. Briefly, 5-d old seedlings grown vertically were transferred to fresh medium containing the indicated compound (Sigma-Aldrich) at the specified concentration. After additional 1 and 3 weeks of growth, PR elongation and shoot biomass/chlorophyll content, respectively, were evaluated. All assays were performed in a climate-controlled growth cabinet under the long-day conditions described above. Similar results were obtained in at least three independent experiments performed with different seed batches (one of which representing a line homozygous for the transgene insertion), and representative data from a single experiment are presented.

### Microscopy

Confocal images were taken with an LSM 510 laser-scanning microscope equipped with a Meta detector (Zeiss, Germany). Excitation wavelengths used to detect fluorescence were 488 nm for GFP, 514 nm for YFP and 543 nm for mCherry. Emitted fluorescence was monitored at detection wavelengths between 565–615 nm for mCherry, between 535–590 nm for YFP and between 500–550 nm for GFP.

### 2,4-D accumulation assays

Radiolabeled [14C]2,4-D accumulation was assayed in root-tip segments as previously described^5, 32^, except that the radiolabeled compound was used at a final concentration of 7.5 μM (50 mCi/mmol) (American Radiolabeled Chemicals).

### Metal ion content determination

To measure total ion content, pooled shoot tissues from 3-week old seedlings challenged with 0.5 mM Al^3+^, 50 µM Cd^2+^, 75 µM Co^2+^, 50 µM Cu^2+^ and 100 µM Ni^2+^ were processed as previously described^48^. Concentration of the digests for Al^3+^, Cd^2+^, Co^2+^, Cu^2+^ and Ni^2+^ was quantified using the Atomic Emission Spectrometry – Inductively Coupled Plasma —Optical Emission System (Perkin-Elmer Optical Emission, *Optima 2100 DV*) at the Laboratório de Análises, Instituto Superior Técnico (Lisbon, Portugal) according to method SMEWW 3120 B described by Eaton *et al*.^54^. Standards for analytical calibration were from Merck KGaA (Germany), and four independent samples were processed per genotype.

### Data Availability

All data generated or analysed during this study are included in this published article (and its Supplementary Information files).

## Electronic supplementary material

Supplementary Information
