# Supplementary material for: Heterologous expression of the yeast Tpo1p or Pdr5p membrane transporters in Arabidopsis confers plant xenobiotic tolerance
Source: Sci Rep. 2017 Jul 3;7:4529. doi: 10.1038/s41598-017-04534-7 (PMC5495770; doi:10.1038/s41598-017-04534-7)
Supplement: Supplementary file 1 — Supplementary Information [file 41598_2017_4534_MOESM1_ESM.pdf]

Supplementary Information

Heterologous expression of the yeast Tpo1p or Pdr5p membrane transporters in Arabidopsis confers plant xenobiotic tolerance

Remy E, Niño-González M, Godinho CP, Cabrito TR, Teixeira MC, Sá-Correia I & Duque P

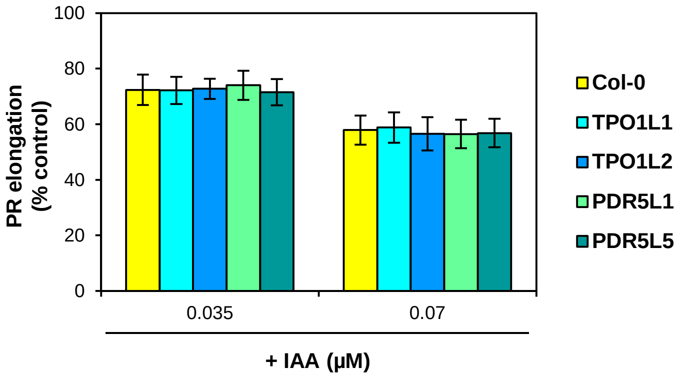

Supplementary Figure S1. Effect of auxin (IAA) on transgenic Arabidopsis lines expressing the yeast Tpo1p or Pdr5p transporters. Effect of the exogenous application of indole-acetic acid (IAA) on PR elongation of seedlings of the wild type (Col-0) and TPO1 (TPO1L1, L2) or PDR5 (PDR5L1, L5) transgenic lines. Values represent means ± SD (n = 8), with similar results being obtained in three independent experiments performed with different seed batches. No statistically significant differences were detected between *ScTPO1*- or *ScPDR5*-expressing lines and the wild type (P>0.5; Student's t-test).

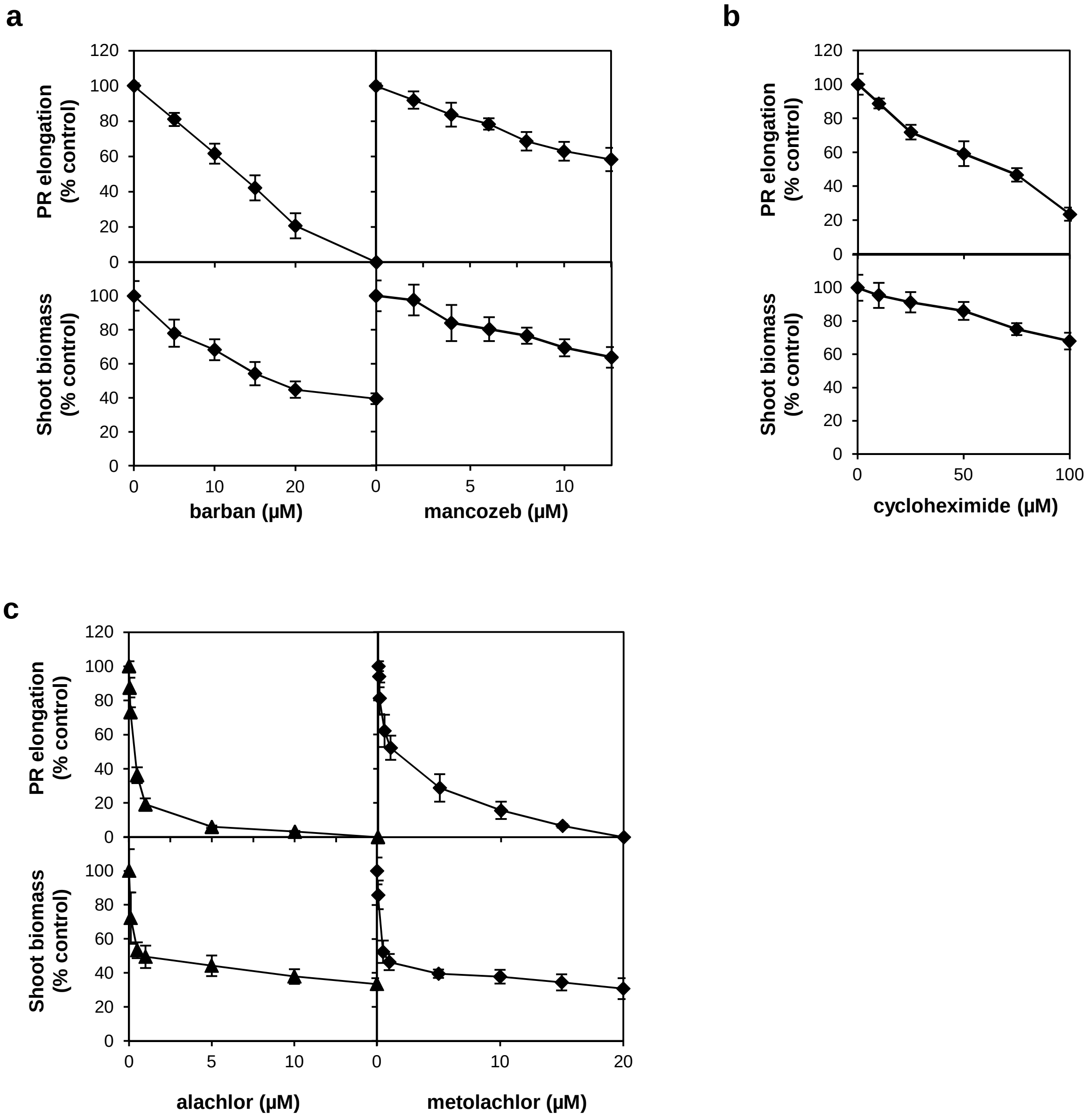

Supplementary Figure S2. Effect of different agricultural pesticides on the growth of wild-type Arabidopsis seedlings. Effect of exogenous application of (**a**) the herbicide barban and the fungicide mancozeb, (**b**) the fungicide cycloheximide and (**c**) the herbicides alachlor and metolachlor on PR elongation and shoot biomass of seedlings of wild-type (Col-0) plants. Values represent means ± SD (*n* = 8), with similar results being obtained in three independent experiments performed with different seed batches.

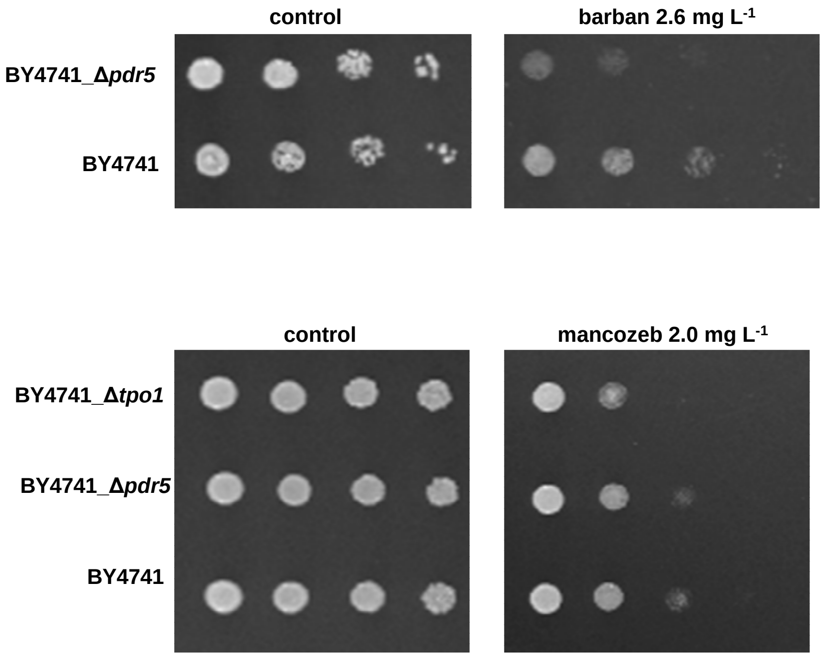

Supplementary Figure S3. Effect of loss of Pdr5p function on susceptibility of yeast cells to a barban or mancozeb challenge. Susceptibility of wild-type and Δ*pdr5* yeast cells treated with 2.6 mg L^{-1} barban, and of wild-type, Δ*pdr5* and Δ*tpo1* yeast cells treated with 2.0 mg L^{-1} mancozeb, analysed through spotting dilution series of cell suspensions (four 1:5 serial dilutions). Similar results were obtained in three independent experiments.

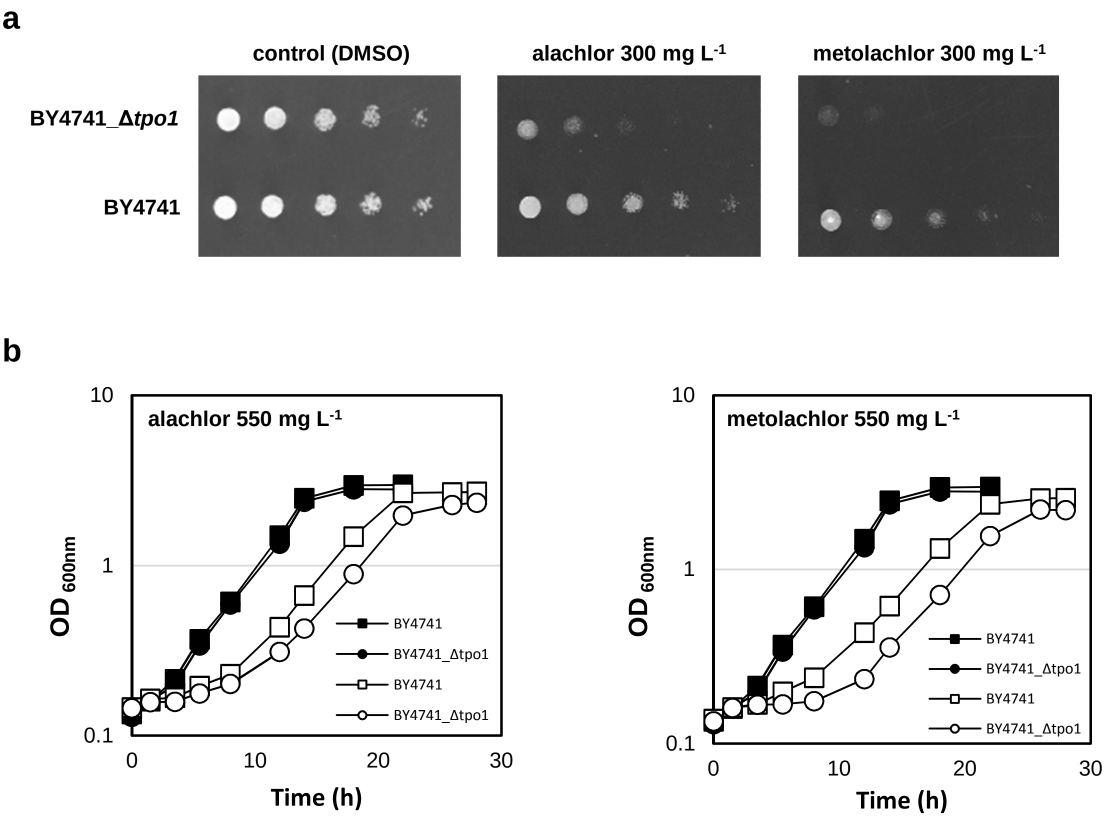

Supplementary Figure S4. Effect of loss of Tpo1p function on susceptibility of yeast cells to an alachlor or metolachlor challenge. (**a**) Susceptibility of wild-type and mutant Δ*tpo1* yeast cells treated with 300 mg L^{-1} alachlor or 300 mg L^{-1} metolachlor, analysed through spotting dilution series of cell suspensions (four 1:5 serial dilutions). (**b**) Comparison of the growth curves of nonadapted wild-type or Δ*tpo1* yeast cells in liquid medium unsupplemented or supplemented with 550 mg L^{-1} alachlor or 550 mg L^{-1} metolachlor. Similar results were obtained in three independent experiments.

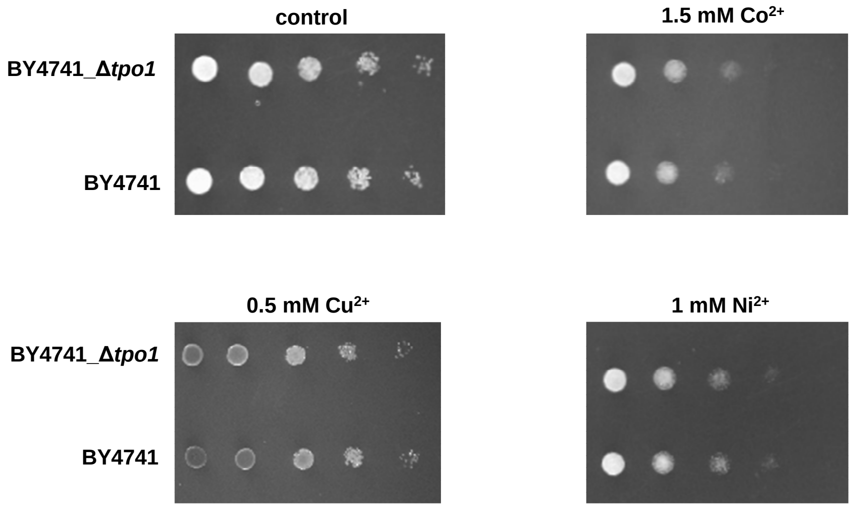

Supplementary Figure S5. Effect of loss of Tpo1p function on susceptibility of yeast cells to toxic concentrations of cations. Susceptibility of wild-type and mutant Δ*tpo1* yeast cells treated with 1.5 mM Co^{2+}, 0.5 mM Cu^{2+} or 1 mM Ni^{2+}, analysed through spotting dilution series of cell suspensions (four 1:5 serial dilutions). Similar results were obtained in three independent experiments.

Supplementary Table S1. Sequences of the primers used.

PRIMER NAME	SEQUENCE (5' to 3')
**Cloning *Pro35S:ScTPO1* construct**	
*Sc*TPO1F	TT*CTCGAG*ATGTCGGATCATTCTCCCATTTCTA
*Sc*TPO1R	TT*ACTAGT*TTAAGCGGCGTAAGCATACTTGGA
**Cloning *Pro35S:ScPDR5* construct**	
*Sc*PDR5F	TT*CTCGAG*ATGCCCGAGGCCAAGCTTAACAA
*Sc*PDR5R	TT*ACTAGT*TTATTTCTTGGAGAGTTTACCGTTC
**Expression analyses**	
RT-PCR	
TPO1F[a]	CTGGTTTTATCTGCGTTTGG
PDR5F[b]	GGGGTGCTTTATTTTGGT
UBQ10F	GATCTTTGCCGGAAAACAATTGG
UBQ10R	TAGAAAGAAAGAGATAACAGG
Real-time RT-PCR	
TPO1qF	TGCCATCGTATTGTTCTTT
TPO1qR	CCCCAGTTATTACTTTGCTT
PDR5qF	GGCAAATCATGGTCAAGCAA
PDR5qR	GCCCAAGTCGCCAAAGTAG
UBQ10qF	GACAATCACCCTCGAGGTGG
UBQ10qR	CATCCTCTAGCTGCTTGCCG
**Subcellular localization studies**	
*Sc*TPO1PacIR[c]	*TTAATTAA*AGCGGCGTAAGCATACTTGGA
*Sc*PDR5AvrIIR[d]	TT*CCTAGG*TTCTTGGAGAGTTTACCGTTC

Restriction sites are shown in italics and underlined.

[a] used with *Sc*TPO1R

[b] used with *Sc*PDR5R

[c] used with *Sc*TPO1F

[d] used with *Sc*PDR5F
